# A Comprehensive Review of Genetically Engineered Mouse Models for Prader-Willi Syndrome Research

**DOI:** 10.3390/ijms22073613

**Published:** 2021-03-31

**Authors:** Delf-Magnus Kummerfeld, Carsten A. Raabe, Juergen Brosius, Dingding Mo, Boris V. Skryabin, Timofey S. Rozhdestvensky

**Affiliations:** 1 Medical Faculty, Core Facility Transgenic Animal and Genetic Engineering Models (TRAM), University of Muenster, Von-Esmarch-Str. 56, D-48149 Muenster, Germany; delf-magnus.kummerfeld@ukmuenster.de; 2 Research Group Regulatory Mechanisms of Inflammation, Institute of Medical Biochemistry (ZMBE), University of Muenster, Von-Esmarch-Str. 56, D-48149 Muenster, Germany; raabec@uni-muenster.de; 3 Institute of Experimental Pathology (ZMBE), University of Muenster, Von-Esmarch-Str. 56, D-48149 Muenster, Germany; RNA.world@uni-muenster.de; 4 Institutes for Systems Genetics, West China Hospital, Sichuan University, Chengdu 610041, China; 5 School of Chemical Biology and Biotechnology, Peking University Shenzhen Graduate School, Shenzhen 518055, China; modingding@163.com

**Keywords:** Prader-Willi syndrome (PWS), Snord116, mouse models, Magel2, PWS imprinting center (IC), non-coding RNAs

## Abstract

Prader-Willi syndrome (PWS) is a neurogenetic multifactorial disorder caused by the deletion or inactivation of paternally imprinted genes on human chromosome 15q11-q13. The affected homologous locus is on mouse chromosome 7C. The positional conservation and organization of genes including the imprinting pattern between mice and men implies similar physiological functions of this locus. Therefore, considerable efforts to recreate the pathogenesis of PWS have been accomplished in mouse models. We provide a summary of different mouse models that were generated for the analysis of PWS and discuss their impact on our current understanding of corresponding genes, their putative functions and the pathogenesis of PWS. Murine models of PWS unveiled the contribution of each affected gene to this multi-facetted disease, and also enabled the establishment of the minimal critical genomic region (*PWScr*) responsible for core symptoms, highlighting the importance of non-protein coding genes in the PWS locus. Although the underlying disease-causing mechanisms of PWS remain widely unresolved and existing mouse models do not fully capture the entire spectrum of the human PWS disorder, continuous improvements of genetically engineered mouse models have proven to be very powerful and valuable tools in PWS research.

## 1. Prader-Willi Syndrome

**Figure 1:**
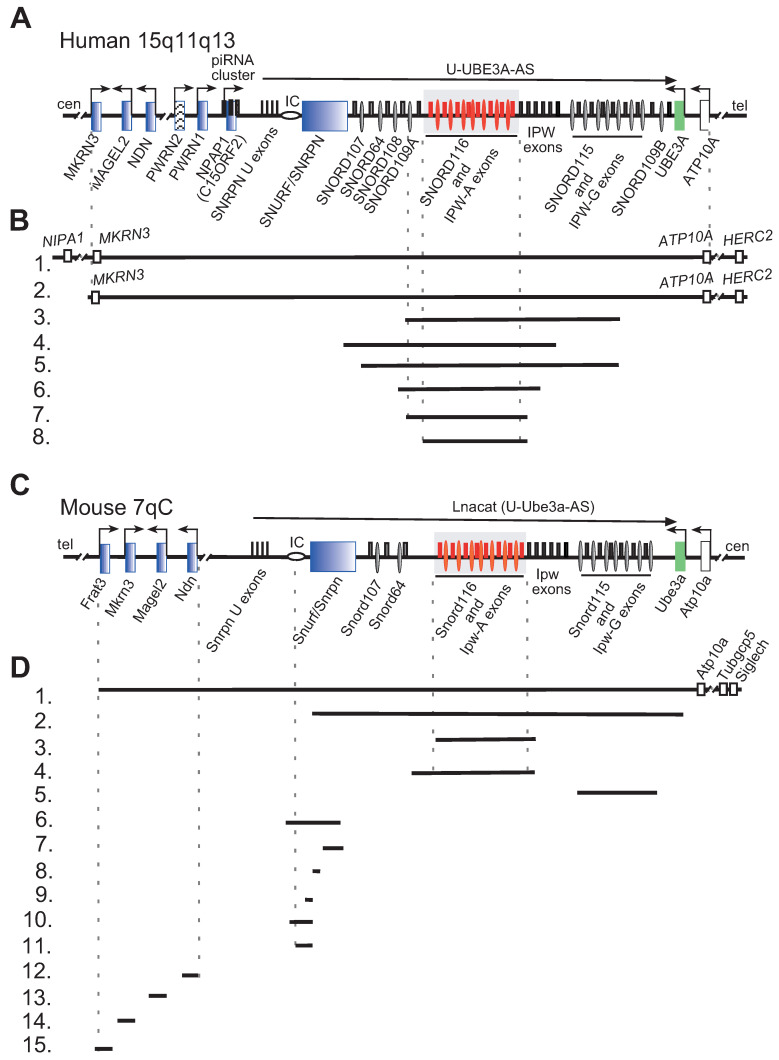
Organization of human and mouse PWS loci, deletions in human and PWS mouse models are indicated. (**A**) Schematic representation of the human PWS locus on chromosome 15q11-q13. Blue rectangles denote paternally imprinted protein coding genes. Thin ovals show snoRNA gene locations; the imprinting center (IC) is denoted by a horizontal oval. Thin rectangles above the midline depict non-protein coding exons. SNORD116 and IPW-A exons are displayed in red and further highlighted by a grey rectangle. Arrows indicate promoters and the direction of transcription. The long arrow on top shows the putative U-UBE3A antisense transcript harboring the SNORD116 and SNORD115 clusters. Centromere and telomere regions are indicated as cen and tel. (**B**) Schematic representation of PWS chromosomal deletions. Lines **1** and **2** indicate the common 5–6 Mb PWS deletion [2]. Lines **3**–**8** represent the characterized PWS cases with microdeletion in the Snord116 array. Line **3**.—[34], **4**.—[35], **5**.—[36], **6**.—[37], **7**.—[38], **8**.—[39] (**C**) Schematic representation of the mouse PWS-locus on chromosome 7qC (symbols as above). (**D**) Schematic representation of available mouse models in PWS research. (**1**.) The largest chromosomal deletion that eliminates the PWS/AS region and a large portion of non-imprinted genes [40]. (**2**.) Deletion of the mouse PWS-locus span from the *Snurf*/*Snrpn* to *Ube3a* genes [41]. (**3**.) Deletion of the PWS critical region (~300 kb) (*PWScr*) comprising of *Snord116* and *IPW-A* gene arrays [42]. (**4**.) The *Snord116del* mouse model eliminating a larger ~350 kb *PWScr* genomic region [43]. Note, that the genomic assembly of *PWScr* is not completed, a gap of ~50 kb inside the Snord116 cluster might increase the snoRNA gene copy number and overall size of deletion (UCSC, GRCm39/mm39 chr7:59457067- 59507068). (**5**.) Deletion of the *Snord115* gene cluster [44] (**6.**–**11**.) Deletions within the *Snurf/Snrpn* and PWS IC center (Details in Figure 2). (**6.**) 35 kb deletion of IC center and *Snurf/Snrpn* exons 1–6 [45]. (**7**.) Deletion of *Snurf/Snrpn* exon 6 including parts of exons 5 and 7 [45]. (**8**.) Deletion of *Snurf/Snprn* exon 2 [41]. (**9.**–**11**.) Elimination of *Snurf/Snrpn* exon 1 and upstream genomic region: 0.9 kb (**8**), 4.8 kb (**9**) and 6 kb (**10**) deletions, respectively [46,47]. (**12**.–**15.**) Deletion of protein coding genes within the PWS-locus: (**12.**) *Ndn* [48,49,50,51]; (**13.**) *Magel2* [52,53]; (**14**.) *Mkrn3* [54]; (**15.**) *Frat3* [30].

Prader-Willi syndrome (PWS; MIM#176270, https://www.omim.org/entry/176270, accessed on 20 March 2021) is a rare, neurodevelopmental, multifactorial genetic disorder resulting from the deletion or silencing of imprinted genes on paternally inherited chromosome 15q11–q13 [1,2,3]. PWS is chiefly caused by a large *de novo* deletion on chromosome 15q11–q13 (~60–70% of cases) (Figure 1A,B(1.,2.)). Approximately 25–35% of cases are caused by maternal uniparental disomy (i.e., two copies of maternal chromosomes UPD15) [4,5]. Less than 5% of PWS patients display defects of the genomic imprinting center (IC) and cases with sporadic chromosomal rearrangements or translocations were rarely identified [5,6,7].

The complex symptomology of PWS is divided into two main and phenotypically opposing stages. The onset of the first stage takes place during the last trimester of pregnancy and proceeds into infancy until around the ninth month. It is characterized by decreased movement and reduced fetal growth in utero, neonatal hypotonia, feeding difficulties and postnatal failure to thrive. This is followed by a transitional phase lasting about five to eight years with comparatively normal weight gain. The second and final stage begins around age eight and extends into adulthood [4,8]. This stage is dominated by hyperphagia and a general lack of satiety; if uncontrolled, significant weight gain ensues transitioning into morbid obesity accompanied by all associated comorbidity risks. PWS patients suffer from general and continued developmental delay, short statue, small extremities and decreased muscle mass. They are frequently affected by respiratory malfunction symptoms, sleep disorders, hypogonadism, mild mental deficiency, and disruptions of their endocrine axis. Individuals display behavioral abnormalities including temper tantrums, obsessive compulsion and skin picking [4,9,10,11]. PWS is a complex disease, and symptoms vary considerably between patients, depending on the size of the chromosomal deletion [2,12,13]. Its prevalence ranges from 1 in 15,000—30,000 births with no observed difference between sexes or ethnicities [1,14,15,16,17]. PWS has a severe impact on the health and life expectancy of affected individuals, leading to a mortality of about 3% per year (approximately three times higher than that of the normal population). Main causes of death are related to respiratory failure, cardiovascular arrest, gastrointestinal blockage as well as infections, pulmonary embolisms and choking [18,19,20]. Therapeutic interventions focus mainly on infant feeding assistance, growth hormone replacement and endocrine dysfunction compensation as well as the treatment of various comorbidities arising from obesity [21,22,23,24]. After more than six decades of research since PWS was first described in 1956, a causative therapy does not exist (MIM#176270, https://www.omim.org/entry/176270, accessed on 20 March 2021).

## 2. The Prader-Willi Syndrome Locus in Mice and Men

Genomic imprinting is an epigenetic process, which via DNA and histone methylation restricts the expression of affected genes in a parent-of-origin specific manner. From the perspective of genome encoded function, the corresponding genes represent a haploid genotype. Loss of the remaining active allele results in expression defects. Historically, PWS was the first identified and characterized disease caused by an imprinting defect and/or uniparental maternal disomy [25,26]. The PWS genomic region harbors protein coding and non-protein coding genes as well as several regulatory elements that modulate imprinting and gene expression (Figure 1). The genomic structure of the PWS locus is highly conserved in mammals, with the murine PWS region on chromosome 7C being almost identical to that of human on chromosome 15. With the exception of the protein-coding gene *Frat3*, which is present only in mice, and reversely for rodents, no homolog to human *NPAP1* (*C15ORF2*) and non-protein coding *SNORD108* or *SNORD109A-B* genes could be identified (Figure 1) [27,28,29,30,31,32]. The conservation of gene organization and imprinting pattern between mice and humans implies similar physiological functions. Therefore, genetically modified mice can represent appropriate models and tools for the investigation of this disease [33].

## 3. PWS Uniparental Disomy (UPD) Murine Models

Maternal uniparental disomy (UPD) of chromosome 15 is the second most common genetic abnormality associated with PWS and is responsible for approximately 35% of cases [5]. Mice with maternal uniparental disomy of the central region on chromosome 7 constituted the first PWS genetic model [55]. It was generated by crossbreeding animals with X-autosomal translocations of the respective region [55]. Newborn pups exhibited weak suckling activity, failure to thrive and ultimately died within two to eight days following birth. No expression of *Snrpn/Snurf* gene was detectable in the brain of mutant pups, suggesting an imprinting defect [55].

This very first model underlined the importance of the paternal allele in the pathogenesis of PWS in mice and defined future efforts to identify the PWS critical genomic region (Table 1).

## 4. PWS Large Chromosomal Deletion Models

In humans, large deletions on paternal chromosome 15q11.2-q13 were detected in more than 60% of diagnosed PWS cases—indicating that this is the most common underlying cause of the disease [5].

A mouse model with a deletion of the entire *PWS* region was generated more than two decades ago by microinjecting a fragment of an Epstein-Barr Virus Latent Membrane Protein 2A (LMP2A) vector into mouse zygotes (B6×SJL) F1 [40]. The resulting transgene contained a 6.8 Mb long array of ~80 repeated *LMP2A* copies that replaced all imprinted genes in the PWS region (Figure 1C,D; Table 1) [56]. For over four generations, the resulting transgenic *PWS*^∆LMP2A^ (*TgPWS*) mice were bred with C57Bl/6 and subsequently with CD1 wild-type mice for the same number of generations. Finally, one stable viable transgenic mouse line derived from a single founder was established.

For *TgPWS* mice with a maternally inherited modified chromosome, no phenotypic abnormalities were observed. This is in stark contrast to paternal inheritance of the modified locus, which led to failure to thrive with fetal and neonatal growth retardation, reduced movement and irregular respiratory rates. Expression of all imprinted genes from the PWS locus was abolished and mice eventually died within one week of birth due to severe hypoglycaemia [57]. Deregulation of the hepatic Igf (Insulin growth factor) axis and increased concentrations of corticosterone and ghrelin were reported for mutant mice. *TgPWS* mice also displayed elevated levels of pancreatic apoptosis; this, in turn led to reduced α- and β-cell masses and lowered levels of pancreatic islet hormones (i.e., insulin and glucagon) [58]. The comprehensive analysis of this mouse model with a large chromosomal deletion in the PWS-locus convincingly demonstrated that the elimination of imprinted genes causes a large spectrum of PWS-related abnormalities associated within the early postnatal period.

In contrast to human, the elimination of all genes within the PWS-locus resulted in early postnatal lethality in mice, which makes it almost impossible to use this model in the detailed investigations of the complete spectrum of PWS pathogenesis. Undoubtably, however, this mouse model confirmed that these human and mouse loci are functionally similar, which justified the utilization of genetically engineered mouse models to examine PWS-related genes in order to advance our understanding of the human syndrome.

## 5. Frat3

The murine *Peg12*/*Frat3* gene (paternally expressed gene 12/frequently rearranged in advanced T-cell lymphomas 3) has no orthologue in the human PWS locus. The single CDS (Coding Sequence) exon encodes a 283 amino acid long protein, which is implicated in canonical Wnt signaling via binding to GSK-3β [101,102]. A knockout mouse model was generated in the 129/Ola background; most of the *Frat3* coding sequence, i.e., including the start codon, was replaced with a *lacZ*-reporter gene cassette, thereby leaving its original promoter intact. Homozygous *Frat3*^lacZ^ mice lacked any obvious phenotype (Table 1) [30]. Moreover, molecular analysis of *Frat*-deficient mice revealed that it is not an essential component of the canonical Wnt pathway in mammals, and *Frat3* is not involved in the PWS phenotype in mouse models [30].

## 6. Mkrn3

Intronless *Mkrn3* (makorin RING-finger protein 3 [formally *Zfp127*]) encodes a putative E3 ubiquitin ligase of 544 amino acids (Figure 1); it inhibits the hypothalamic–pituitary–gonadal axis, thereby modulating the onset of puberty in mammals [103].

*Mkrn3* knockout mice were generated on the C57BL/6 wild-type background via the introduction of an engineered 2 bp frameshift deletion at position 275–276 of the ORF (Open Reading Frame) in the single CDS exon of the gene (Table 1) [54]. Mating of *Mkrn3*^m+/p−^ males with WT (wild-type) females yielded offspring in the expected Mendelian ratio. Compared to their respective WT littermates of the same sex and age, *Mkrn3*^m+/p−^ male mice were noticeable lighter between postnatal day P15 to P60. The corresponding weight gain for female *Mkrn3*^m+/p−^ mice proceeded biphasically; KO (knockout) mice were heavier between day P15 to P40, but gained less weight compared to age-matched controls from day P45 to P60. Another effect of the *Mkrn3* knockout was the earlier onset of puberty in both sexes, induced by an increased production of hypothalamic GnRH1 (gonadotropin-releasing hormone).

*MKRN3* is an unlikely contributor to the key symptoms of PWS in humans [104]. However, these findings advanced our understanding of general regulatory mechanisms of the pubertal process and putative molecular defects causing PWS-related hypogonadism. Moreover, the genetically engineered *Mkrn3*^m+/p−^ mouse model served as a useful tool to investigate the molecular mechanism underlying human central precocious puberty (CPP) syndrome.

## 7. Magel2

*MAGEL2* (Melanoma Antigen-subfamily like 2) is another paternally imprinted intronless gene, within the PWS locus (Figure 1) [64,72,105]. The human gene encodes a protein of 529-amino acids (525 amino acids in mouse) that displays about 51% sequence similarity to *NECDIN,* and functions as an E3 ubiquitin ligase enhancer involved in retromer endosomal protein trafficking [106,107,108]. *MAGEL2* is particularly interesting, as patients with inactivated mutations of this gene develop the Schaaf-Yang syndrome (SYS; MIM#615547, https://www.omim.org/entry/615547, accessed on 20 March 2021). SYS shares many symptoms with PWS, including neonatal hypotonia, feeding difficulties, developmental delay, hypogonadism, intellectual disability, and a prevalence to autism-related disorder [109,110,111].

For the in vivo investigation of *MAGEL2* functions, C57Bl6-derived *Magel2*^m+/p−^ mice were generated by replacing the C-terminus of the open reading frame with a lacZ reporter (Table 1) [53]. Although the promoter region remained intact, these mice entirely lacked Magel2-LacZ fusion protein expression [53,59]. Subsequent analysis revealed that in addition to Magel2-LacZ, the expression of the Ipw-A non-protein coding transcript was significantly reduced. The *Magel2*^m+/p−^ neonates displayed a postnatal lethality of ~10%, failure to thrive and growth retardation, resulting in a slight decrease in body weight until weaning (~P28). After weaning, mutant mice equaled the body weight of their WT littermates; however, KO mice were characterized by higher body fat percentage as well as decreased lean mass and muscle fibers. In addition, the bone mineral density was decreased [53,59,61,63,64,67]. When *Magel2* KO mice were fed a standard diet for 12–14 weeks, a slight increase in body weight—compared to WT littermates—was observed [59,69]. Glucose and cholesterol homeostasis was impaired in *Magel2* KO mice. In addition, signs of insulin and leptin resistance, defective responses to ghrelin stimulation, higher serum adiponectin concentrations, elevated corticosterones, a malfunctioning growth hormone axis and compromised melanocortin signaling were identified [59,62,64,65,66,112]. Changes in oleoylethanolamide signaling were also reported [69]. Concentrations of several neurotransmitters were lower in *Magel2*-null mouse brains, including those of dopamine and serotonin [60,70]. In addition, the lack of *Magel2* in KO mice was accompanied by reduced levels of several neuropeptides, i.e., oxytocin and orexins. Furthermore, the expression of regulatory proteins that participate in processing and exocytosis of neuropeptides via secretory granules was compromised [53,71,72]. *Magel2* null mice also revealed higher levels of mTOR and its downstream signaling targets in the hypothalamus [68]. The mutant mice displayed different feeding behavior, most notably an increased portion size and time per meal after a 24 h fast [69]. *Magel2*^m+/p−^ mice were on average less active, both in familiar and novel environments [53,61]. They exhibited increased anxiety and impaired social behavior [60,113]. Absence of *Magel2* expression reduced the reproductive fitness in both sexes, results in delayed onset of puberty and irregular estrous cycles in females and decreased olfactory preference for aesthetic female odor as well as significantly reduced levels of serum testosterone in males [60].

An independent KO model for *Magel2* was generated on a 129Sv/Pas genetic background. The entire gene promoter and approximately 75% of the CDS were deleted (Table 1). The resulting mice were subsequently backcrossed to the C57Bl6/J background [52]. *Magel2*^m+/p−^ neonates were not hypotonic, but sustained significantly (~50%) increased lethality, caused by weak or even absent suckling activity. Lack of *Magel2* expression also impaired the production, particularly the processing from precursor to active peptide of hypothalamic neuropeptides, such as oxytocin and orexin-A. *Magel2* mutants also exhibited deficiencies in social behavior and learning abilities [73]. The suckling initiation deficit phenotype as well as the development of behavioral abnormalities could be resolved by the injection of oxytocin in the first postnatal week [52,73].

More than half of SYS patients express a truncated version of *MAGEL2*, which is the result of mutation or deletion within the so-called proline-rich region upstream of the C-terminus [109]. Two additional mouse models were generated to analyze the functional impact of these variants. One represented a similarly truncated version of *Magel2* (*trMagel2*) as detected in SYS patients, and a second overexpressed the transgenic *Magel2* (*CAG*-trMagel2) N-terminal domain (amino acid residues 1–437) under the control of the CAG promoter (Table 1) [74]. Overexpression of the Magel2 truncated protein was highly toxic, as all *CAG*-trMagel positive pups died between birth and postnatal day 13. Heterozygous mice carrying the truncated *trMagel2* on either the paternal *Magel2^m+/p-tr^* or maternal *Magel2^m−tr/p+^* allele had no obvious phenotype. The neonatal *Magel2^m+/p-tr^* pups were lighter than their wild-type littermates but gained weight faster until there was no detectable difference at the age of eight weeks.

Mice lacking *Magel2* recapitulate a basic aspect of PWS and SYS stage I. However, none of the models developed the more severe symptoms of later stages of PWS, severe obesity combined with hyperphagia. Nevertheless, understanding the physiological function of *Magel2*, particularly its role in the proteolytic processing pathways of several hormones, implies an important contribution to many symptoms associated with PWS and SYS.

## 8. Necdin

The intronless Necdin (*NDN*) gene encodes a 321 amino acids long protein which is a member of the MAGE family and implicated in cell survival, maintenance of circadian rhythm [80], neuron migration and growth [114,115]. Neurological symptoms in PWS patients suggested a functional role of *NDN* in the pathophysiology of the disease [116]. To analyze *Ndn* functions in vivo, several knockout mouse models were generated. The first model was engineered in the 129SV mouse background (Table 1). The entire CDS and promoter region of *Ndn* was replaced by a ß-Galactosidase reporter [51]. The resulting mice were null for *Ndn* expression. Both homo- and heterozygous mutants were viable, had normal fertility, showed the same body weight development as WT mice and did not develop obesity until 10 months of age.

In-frame fusion of the first 31 *Ndn* codons with a *lacZ* reporter and replacement of the downstream CDS with the cassette was used to generate a second KO model in 129Sv derived ES (Embryonic stem) cells (Table 1). The KO strategy left the endogenous promoter intact; the resulting chimeric males were crossed with C57BL/6 WT females [48]. Depending on the genetic background of the female, the resulting heterozygous offspring displayed varying degrees of postnatal lethality due to respiratory problems from 80–95% for C57BL/6 and 25% for FVB to WT levels for (C57BL/6 × C3H)F1 hybrids. In most cases, the lethality was higher in male than female offspring. Respiratory distress originated from abnormalities in serotonergic modulation of the respiratory rhythm generating neurons, which are particularly sensitive to loss of *Ndn* expression [75,76]. The surviving mice were not associated with any detectable phenotype, did not develop obesity until 10 months of age and were fertile.

The third *Ndn* KO model was generated by replacing the promoter and two-thirds of the N-terminal *Ndn* coding sequence with the *Neo* cassette flanked by *LoxP* sites, which was subsequently removed by crossing with *Cre*-expressing C57Bl6/J females (Table 1) [50]. Heterozygous neonates displayed 21–31% lethality due to respiratory distress [50,67]. The surviving mice were fertile, exhibited normal weight development and did not become obese during an 18-month observational period. *Ndn*^m+/p−^ mice displayed a significant reduction in oxytocin-producing and luteinizing hormone-releasing hormone (LHRH)- producing neurons in hypothalamus. The *Ndn* null mutants had a normal circadian rhythm, normal motoric skills and showed no abnormalities in anxiety-related behavior, but were superior in spatial learning and memory tests. Notably, skin scraping was significantly increased in *Ndn*^m+/p−^ mice compared to the wild-type control. Homozygous *Ndn*^m−/p−^ offspring had an even higher postnatal lethality of 43% compared to heterozygotes, most likely due to respiratory deficiency [77]. Interestingly, low *Ndn* expression levels were detectable from the maternal allele in two *Ndn*^m+/p−^ mouse strains (C57Bl/6J and 129Sv/Pas). There was also a drastic (i.e., up to three orders of magnitude) inter-individual variability of *Ndn* expression between *Ndn*^m+/p−^ mice. Complete loss of *Ndn* expression in surviving homozygous mice altered the development and function of serotonergic neurons, resulting in central apnea and hypercapnia [78]. *Ndn*^m+/p−^ mice showed a significant, i.e., up to three-fold, upregulation of *Slc6a4* (serotonin transporter, solute carrier family 6 member 4) expression, which results in an increase of serotonin (re-)uptake and thereby decreased extracellular serotonin to insufficient levels, ultimately causing breathing deficits.

Another model was generated by conventional gene targeting, with a 1.5 kb *Pgk*/*NeoR* cassette inserted into the *Ndn* gene to disrupt its coding sequence (Table 1) [49]. The targeting vector was electroporated into TT2 ES cells. Positive clones were injected into ICR embryos and the resulting chimeras were backcrossed to ICR wild-type mice. The heterozygous *Ndn*^m+/p−^ mice did not reveal any increased postnatal lethality, were fertile and bred in the normal Mendelian ratio. Necdin deficiency triggered apoptosis in developing mouse dorsal root ganglia at E12,5 and led to a significant reduction in total neuron number at P0. The *Ndn*^m+/p−^ also had a significantly increased tolerance to pain compared to the wild-type control at the age of two and four weeks. Five-day-old *Ndn*^m+/p−^ neonates displayed some signs of hypotonia, although there were no significant differences by the age of two weeks [79]. The mutant also had an impaired ventilatory response to hypercapnia at postnatal days 4 and 8. The neurons of the noradrenergic system in the locus coeruleus of *Ndn*^m+/p−^ mice are characterized by decreased spontaneous activities and showed impaired excitability.

Recently, using CRISPR/Cas9-mediated genome engineering, a Necdin deficient mouse model harboring a 1349 bp deletion within the *Ndn* gene was generated on a C57BL/6 background (Table 1) [80]. Lack of necdin protein destabilized a key component of the circadian clock and resulted in alterations of clock gene expression compared to wild-type and an unstable circadian rhythm in Ndn^m+/p−^ mice.

The *Ndn* mouse models successfully reproduced both the respiratory and sleep disorder related phenotype of PWS; therefore, they are suitable for the development of novel therapeutic approaches, as respiratory failure is the most common cause of death in infants and children diagnosed with PWS [18]. As previously noted [117], it is remarkable that the majority of protein coding genes in this locus were generated via retrotransposition, emphasizing once more the significance of this mechanism for the evolution of genes and entire gene loci [118].

## 9. Snurf/Snrpn

**Figure 2:**
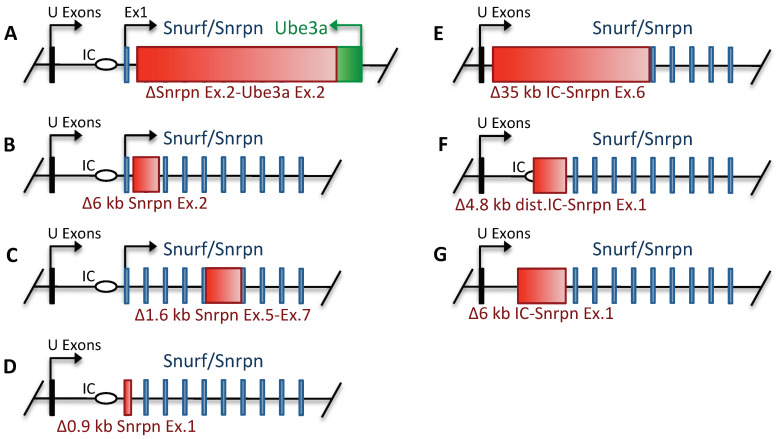
Schematic representation of genetically engineered mouse models harboring deletions within *Snurf/Snrpn* and PWS IC center (drawings are not to scale). Green and thin blue rectangles denote *Ube3a* and *Snurf/Snrpn* CDS exons, respectively. The red rectangles indicate generated genomic deletions. Thin black rectangles show location of U-exons; the imprinting center (IC) is denoted by a horizontal oval. (**A**). Large deletion of the genomic region between *Snurf/Snrpn* exon 2 and *Ube3a* exon 2. [41]. (**B**). Inactivation of the Snurf ORF by deleting the 6 kb region including *Snurf/Snrpn* exon 2 [41]. (**C**). Intragenic deletion of 1.6 kb within *Snurf/Snrpn* including exon 6 and parts of exons 5 and 7 disrupting the Snrpn ORF [45]. (**D**). Small 0.9 kb deletion of the major *Snurf/Snrpn* promoter together with first CDS exon [46]. (**E**). The PWS IC deletion, spanning 35 kb (originally described as 42 kb) including *Snurf/Snrpn* exons 1–6 [45]. (**F**). Deletion of 4.8 kb (later revealed to be 5.07 kb in size) genomic region, including *Snurf/Snrpn* exon 1 and the distal part of the PWS IC [46]. (**G**). The 6 kb deletion comprising PWS IC and *Snurf/Snrpn* exon 1 [47].

The *SNURF/SNRPN* (Small Nuclear Ribonucleoprotein Polypeptide N) gene encodes two different proteins from a bicistronic transcript spanning 10 exons and is highly conserved between humans and mice [119]. CDS exons 1–3 encode for the 71 amino acids protein Snurf (SNRPN upstream open reading frame), while CDS exons 4–10 encode for a 240 amino acids component of the SmN complex (small nuclear ribonucleoprotein complex) [120]. The SNURF/SNRPN bicistronic transcript is highly expressed in the brain and heart in both humans and mice. SNURF localizes to the nucleus but its function is still unknown. SmN plays a role in pre-mRNA processing and possibly alternative splicing, regulating the development of the spine and cerebral cortex in mice [121,122,123]. For numerous patients diagnosed with PWS, deletions of the *SNURF/SNRPN* gene were detectable, suggesting that its product(s) might contribute to the pathology of the disease [124,125,126]. For the analysis of *Snurf*/*Snrpn* functions, seven different genetically modified mouse models were generated (Figure 2). Three of them contained small deletions of the *Snurf/Snrpn* protein coding region, eliminating CDS exon 2, exons 5–7 and exon 1, respectively (Figure 2B–D). A fourth model harbored a long deletion eliminating *Snurf/Snrpn* exon 2 up to the *Ube3a* gene region (Figure 2A) [41,45]. Regardless of the actual mouse genetic background, i.e., C57BL/6 or 129/SvEv–C57BL/6J hybrids, the offspring with paternally inherited *Snurf/Snrpn* small deletions (Figure 2B–D) were indistinguishable from wild-type littermates. They exhibited a normal imprinting pattern, were fertile and displayed no obvious phenotypic abnormalities [41,45]

Different results were obtained in a mouse model with a deletion from Snrpn exon 2 to *Ube3a* (Figure 1D and Figure 2A; Table 1) [41]. Mice homozygous for this long deletion did not survive past E20. Heterozygotes with the long deletion on the paternal allele were weak, hypotonic and had weak suckling activity. They were underweight at birth and showed severe growth retardation compared to WT littermates; only about 20% of the mutant pups survived until weaning. The surviving knockout mice were fertile, had about 2/3 of the bodyweight of age-matched WT animals, and did not develop obesity within a 14-month observational period [41]. These data raised doubts with regards to the functional significance of the *SNURF/SNRPN* gene as the primary cause of PWS and underlined the importance of the *PWS* critical region, which at the time, had not been identified.

## 10. PWS Imprinting Center

In the late nineties, a 35 kb deletion (originally reported as 42 kb) encompassing *SNURF/SNRPN* CDS exons 1–6 and its 16 kb upstream region was generated in C57BL/6 mice by replacing the corresponding sequence with a Pgk-Neo-polyA cassette (Figure 1D and Figure 2E; Table 1) [45]. This deletion (*PWS-IC^∆^*^35kb^) resulted in a complete imprinting defect and thus abolished expression of all imprinted genes in the locus. *PWS-IC^∆^*^35kb^ neonates had decreased body weight and hypotonia at birth, resulting in poor suckling and low blood glucose levels. Ultimately, the offspring died within the first week due to feeding difficulties. However, this fully penetrant neonatal lethality was highly dependent on the genetic background. C57BL/6 males carrying the maternal *PWS-IC^∆^*^35kb^ allele produced viable offspring (survival rates between 10%–60%) with female mice of FVB/NJ, C3H/HeJ, 129S1/Sv and BALB/cJ genetic background, but not with C57BL/6J or DBA/2J animals [81]. Viable pups, especially after separation from WT siblings, grew into adulthood and were at all times smaller than WT controls. *PWS-IC^∆^*^35kb^ had normal fertility and did not develop obesity until the end of the 15-week observational period. The mouse background-dependent survival was putatively independent of residual expression of genes from the paternal chromosome or leakage from the maternal chromosome, since the detected expression levels did not correlate with survival rate [81]. The most likely explanation of the observed differences is strain specific gene-modifiers affecting survival [81]. *PWS-IC*^m+/p∆35kb^ mice generated by crossing *PWS-IC^∆^*^35kb^ C57BL/6J males to CD-1 females exhibited less locomotive activity and impaired attentional functions, but normal anxiety levels compared to WT [82]. Furthermore, *PWS-IC*^m+/p∆35kb^ mice showed increased impulsivity and locomotor activity when motivated by food reward [83]. There were no detectable differences in whole tissue monoamine levels or expression and splicing of 5Ht2cr (see below). The *PWS-IC^m^*^+/p∆35kb^ mice also underperformed in a stop-signal reaction time task compared to WT mice suggesting increased impulsivity. This effect could be ameliorated with the selective 5Ht2cr agonist WAY163909 [85]. Compared to the wild-type control, circulating ghrelin levels were elevated up to threefold in *PWS-IC*^m+/p∆35kb^ mice. Mutants were also characterized by significantly higher food consumption, both with ad libitum access and after an overnight fast [84]. In contrast to wild-type mice, *PWS-IC*^m+/p∆35kb^ mice reacted with apathy towards non-caloric sweetener and showed a preference for food of high caloric value. Interestingly, *PWS-IC*^m+/p∆35kb^ mice performed better in a maze-learning test than wild-type mice when combined with food reward. These findings might suggest increased motivation due to stronger food-seeking behavior [86].

To narrow down the critical size of the imprinting center, additional in vivo models were generated by targeting the DNA methylation region (DMR) located upstream of the *Snurf/Snrpn* CDS exons. Two models encompassing deletions of 0.9 kb and 4.8 kb were generated (Figure 2D,F; Table 1). The latter was later revealed to be 5.07 kb [47]; but, designated as *PWS-IC*^m+/p∆4.8kb^ model in the literature. Notably, the larger deletion eliminated almost the entire methylation region (~2.7 kb), leaving only a small part intact [46]. Heterozygous mice harboring the small deletion (0.9 kb) displayed a regular imprinting pattern, were phenotypically unremarkable, fertile and had no weight abnormalities compared to WT mice. The paternal inheritance of the 4.8 kb deletion, on the other hand, resulted in an approximate 40% postnatal lethality. The growth of surviving *PWS-IC*^m+/p∆4.8kb^ mice was retarded, amounting to a 30% decrease in size and bodyweight. However, the mutant mice harboring the paternal deletion were fertile. Notably, the 4.8 kb deletion resulted in a partial imprinting defect, therefore the *PWS-IC*^m+/p∆4.8kb^ mice displayed low expression levels of *PWS*-locus encoded genes.

To define the imprinting center of the mouse PWS-locus, a further model harboring a 6 kb deletion was generated, eliminating about 3.7 kb upstream from *Snurf/Snrpn* exon 1 (Figure 2G; Table 1) [47]. The 3′ end of this 6 kb deletion was identical to that of the previously mentioned 4.8 kb deletion. The *PWS-IC*^m+/p∆6kb^ offspring containing the deletion on the paternally inherited chromosome were significantly smaller and weaker than their WT littermates. They often lacked milk spots and did not survive past postnatal day 7, with most of the pups dying within 48 h after birth. *PWS-IC*^m+/p∆6kb^ mice displayed no detectable expression of PWS genes from the paternal allele, indicating that functional elements enabling *PWS-IC* activity were present within the deleted interval [47].

For the investigation of the human PWS imprinting region, a knock-in transgenic mouse model was generated by replacing the mouse 6 kb *PWS-IC* with a 6.9 kb fragment of the entire human *PWS-IC* region (Table 1) [47,87]. Knock-in mice harboring human *PWS-IC*^Hs^ acquired maternal DNA methylation patterns. Paternal inheritance of the *PWS-IC*^Hs^ led to a neonatal lethality of 47% in the second generation with a C57BL/6J background and 16% postnatal lethality with a 129S1/Sv background. The *PWS-IC*^Hs^ mutant pups were significantly smaller, had difficulties feeding and displayed considerable growth retardation, which was accompanied by a 50% reduced bodyweight; the effect persisted into adulthood. Paternal inheritance of the *PWS-IC*^Hs^ resulted in almost complete absence of the *Ndn*, *Mkrn3*, *Magel2* and *Frat3* gene products, but expression of U-Ube3a-As derived non-protein coding RNAs, including Snord64, Snord116, Snord115 and Ube3a-As remained unaltered [87].

The data implied that the mechanism to acquire silencing is conserved between humans and mice, but the maintenance and regulation of the silenced state is different. This also might explain the differences in the regulation of tissue-specific expression of PWS encoded genes between both species [127]. In any event, the analysis conducted with these mouse models defined the PWS-locus imprinting center in mice and revealed that the imprinting effect is similar in mice and men.

## 11. Snord116 Gene Cluster

Imprinted SNORD small nucleolar RNAs (snoRNAs) within the PWS-locus were originally identified two decades ago [128]. Based on the presence of conserved C and D sequence motifs, these snoRNAs were assigned to the subclass of 2′-O-methylation guide C/D box snoRNAs. However, due to the apparent lack of any significant complementarities to classical snoRNA target molecules (rRNAs, snRNA) they are referred to as “orphan” snoRNAs [129]. The human PWS region harbors seven *SNORD* genes and families: *SNORD107*, *SNORD64*, *SNORD108*, *SNORD109A*, *SNORD116*, *SNORD115* and *SNORD109B*. The corresponding mouse locus contains the orthologous genes *Snord107*, *Snord64*, *Snord116* and *Snord115*. Most snoRNAs from the PWS-locus are processed from introns of a long primary non-protein coding U-UBE3A-AS transcript (U-Ube3a-AS in mouse) (Figure 1). Both the human and murine genes are paternally imprinted. In human, SNORD116 and SNORD115 genes represent tandemly repeated arrays comprised of 29 and 48 copies, respectively [129]. *SNORD116* copies are located within introns flanked by the repetitive *IPW-A* exons of the *U-UBE3A-AS* transcript (Figure 1A). *SNORD115* copies are embedded between repetitive *IPW-G* exons (Figure 1A). In mice, 66 copies of *Snord116* are distributed in the introns of 67 *Ipw-A* exons (Figure 1C). However, due to an assembly gap of approximately 50 kb inside the mouse *Snord116* region, the exact number of repeats is yet to be determined. Although *SNORD116* displays a high degree of sequence similarity between different mammalian species [130], conventional targets on rRNAs cannot be identified [128,129]. In rodents, expression of the PWS locus encoded snoRNAs is restricted to neurons, while in humans they are most abundant in brain but also expressed in other tissues [127].

### 11.1. The First Mouse Model Harboring the Deletion of the PWS Critical Region (PWScrm+/P−)

Analysis of the aforementioned mouse models that abolished the expression of single or multiple genes predicted PWS critical region (*PWScr*) within the *Snord116* gene cluster [131]. To investigate the putative contribution of *Snord116* to the PWS phenotype in vivo, two mouse models were generated. The first model harbored a ~300 kb genomic deletion (UCSC, GRCm39/mm39 chr7:59,277,590-59,580,881) of the *Snord116* and *Ipw-A* gene arrays and was designated as *PWScr* deletion model (Figure 1 C,D; Table 1) [42]. *PWScr*^m+/p−^ pups displayed significant growth retardation starting from postnatal day 5 lasting into adulthood, i.e., up to one year of monitoring. Growth retardation was observed independent of the following genetic backgrounds: 129SV × C57BL/6 (>85% C57BL/6 contribution), 129SV × C57BL/6 × FVB/N (~50% FVB/N contribution) as well as 129SV × C57BL/6 × BALB/c (~50% BALB/c contribution). A slight increase in postnatal lethality of about 15% as well as between P1 and P22 was observed for the 129SV × C57BL/6 genetic background. Because no difference in bodyweight was detected for embryos at E12.5, E15.5 or E18.5, the failure to thrive was most likely caused by reduced feeding capabilities. *PWScr*^m+/p−^ mice were fertile, bred in the expected Mendelian ratio and did not become obese at any point in time [42]. Expression of other genes in the PWS locus remained unaffected, except for a small decrease of the *Snord115* and *Ipw-G* exon expression [97].

In addition, magnetic resonance imaging revealed a decrease of grey-matter volume in the ventral hippocampus and septum areas of *PWScr*^m+/p−^ mice [89]. Orexin and melanin concentrating hormone systems in the lateral hippocampus were impaired in mutant mice, as the deletion of *Snord116* gene cluster causes a 60% reduction in orexin expressing neurons [88]. Consequently, expression of *pOx* (prepro-orexin) and *Peg3* (paternally imprinted gene 3) were significantly upregulated in *PWScr*^m+/p−^ [88]. The analysis of RNA-seq data led to the identification of >4000 differentially regulated genes in the hypothalamus of *PWScr*^m+/p−^ mice compared to wild-type controls [88]. Among the upregulated genes were those related to neurotransmitter transport, synaptic organization and cytokine production pathways [88]. *PWScr*^m+/p−^ mice also exhibited dysregulated “rapid eye movement” (REM) sleep, reduced peripheral thermoregulatory response, as well as an increase of peripheral body temperature compared to wild-type littermates during the light phase of the day. Those observations suggested that in addition to *Ndn, PWScr* derived non-protein coding RNAs also contribute to the regulation of sleep physiological measures in PWS [88].

### 11.2. The Second Mouse Model Harboring the Deletion of the PWS Critical Region (Snord116del)

The second mouse model featured an ~350 kb deletion of the *Snord116* gene cluster (UCSC, GRCm39/mm39 chr7:59,275,265-59,624,663—based on the location of genotyping primers). This eliminates an ~50 kb longer upstream region as opposed to the *PWScr*^m+/p−^ model (Figure 1C,D; Table 1) [43]. Gene targeting was performed by homologous recombination in BRUCE4 ES cells, thereby introducing *LoxP* sites flanking the *Snord116* gene array. Selected ES cell clones were injected into C57Bl/6J-Tyr^c−2J/J^ albino blastocysts. Heterozygous offspring derived from the chimeras were mated with a transgenic C57BL/6J strain expressing Cre-recombinase. In a second experimental approach, targeted ES cells were transfected with a Cre expressing vector prior to blastocyst injection [43].

Offspring from both lines exhibited a similar phenotype and transmitted the paternal deletion through the germline. Although the entire cluster consisting of *Ipw-A* exons and *Snord116* genes was paternally deleted, the mouse model was named *Snord116del*. The expression of other genes in the *PWS*-locus remained unaffected by the deletion, except for the neighboring genes flanking the deletion. Thus, an approximate 35% decrease and a 33% increase in expression of *Snord107* and *Snord115* genes was observed, respectively [43]. Notably, this is in contrast to a slight decrease of the *Snord115* gene expression in *PWScr*^m+/p−^ mice compared to WT littermates [97]. Similarly, to the *PWScr*^m+/p−^ model, newborn *Snord116del*^m+/p−^ pups P0 harboring the paternally inherited deletion were indistinguishable from WT littermates. Growth retardation was detected from postnatal day 2 onwards.

In *Snord116del* mice, which were homozygous for the deletion (*Snord116del^m−/p−^*), reduced bone and fat mass relative to their bodyweight accompanied by an increase in lean mass was reported [95]. *Snord116del* mice were fertile and bred normally, although the female sexual maturation was delayed. Interestingly, postnatal lethality was not reported for this *Snord116del* model and mice were healthy during the 18-month observation. It was hypothesized that the failure to thrive was due to hypotonia and insufficient suckling, but neither hypotonia nor empty stomachs were observed in *Snord116del* pups. However, the livers and stomachs of *Snord116del* pups weighed less at P5 and P13 than those of their WT littermates, which might indicate a decreased rather than absent milk intake. An overall decreased stomach weight was also observed in the *PWScr*^m+/p−^ model, but the effect was not statistically significant when the reduced bodyweight of the animals was taken into account (Skryabin et al., unpublished). Igf1 levels were significantly lower in mice lacking Snord116 expression, although there were no detectable anomalies of the pituitary gland itself [43].

The onset of locomotive abilities was delayed in *Snord116del* mice; yet, no differences in motor abilities were detected when reflex-related tasks were tested [90]. Furthermore, *Snord116del* mice exhibited an impairment in the recognition of novel objects and the memory of object location. In addition, the mice demonstrated a tendency towards increased anxiety-related behavior.

Food consumption was normal in the paternally deleted *Snord116del ^m+/p−^* mice, both on regular chow and high-fat diet. Indeed, the mutants were even somewhat resistant to obesity, as— compared to WT siblings—they displayed significantly lower bodyfat percentages after 4 months on a high-fat diet [43]. Despite these findings, initial analysis of *Snord116del^m+/p−^* mice revealed an increase in food intake relative to their lower bodyweight, which was interpreted as hyperphagia [43]. Forthcoming studies revealed altered diurnal energy regulation in *Snord116del* mice, thereby showing decreased respiratory exchange rates (a result of increased fat oxidation as opposed to carbohydrate) during the 12-h light period [94]. Ghrelin levels in *Snord116del* with *ad libitum* access to food were significantly increased and comparable to the level observed in WT mice after a 24h fast, whereas insulin sensitivity was normal in mutant females but increased in males [94]. In late adulthood (28–34 weeks of age), increased glucose tolerance and insulin sensitivity were detected independent of *Snord116del^m−/p−^* gender [95]. *Snord116del^m−/p−^* mice exhibited ~11% and ~31% higher calories per gram of bodyweight uptake in early (12–16 weeks of age) and late adulthood (28–34 weeks of age) [95]. In early adulthood, *Snord116del^m−/p−^* mice showed lower activity levels during the 12-h dark phase and increased energy expenditure during the light phase. However, in late adulthood, this profile was inverted, leading to increased activity during the dark phase [95]. Core body temperature was also reduced in mutant mice in early adulthood. *Snord116del^m−/p−^* mice were partially resistant to high fat diet-induced obesity, which subsequently, did not lead to a significant increase in bodyweight compared to normal chow, although the fat mass was increased [95]. In stark contrast, recent analysis uncovered that paternally inherited *Snord116del* mice displayed no significant differences (compared to the WT controls) in the 24-h food intake of animals that had *ad libitum* access or those following a 24-h fast [91]. However, when the Snord116 cluster was specifically eliminated in the mediobasal hypothalamus of adult mice, hyperphagia leading to obesity in a subset of animals was detected [91].

However*,* when the *Snord116del*-based model harboring a mosaic partial deletion of the *Snord116* gene cluster was investigated in adult animals, the 16% reduction of Snord116 expression did not result in any significant effect on bodyweight. In addition, no remarkable change in weight of any major tissue/organ or even the lean mass was identified [96]. Reduction of Snord116 expression resulted in an increase of small white adipose tissue mass; yet, food intake was reduced during *ad libitum* food access or after a 48-h fast [96]. The mosaic mice displayed an impaired glucose clearance rate and insulin resistance at 13 weeks of age [96]. There was no detectable difference in energy expenditure or respiratory exchange rate, only a short time delay during the transition from the light, somnolent phase to the dark, active phase.

Changes in expression profiles of selected genes were investigated in *Snord116del* mice by various research groups. The analysis of genes representing the leptin/melanocortin pathway revealed inconsistent results. Some approaches uncovered no altered expression profiles for *Npy* (neuropeptide Y), *Lepr* (leptin receptor), *Agr* (agouti-related protein), *Pomc* (Proopiomelanocortin), *Pcsk1* (prohormone convertase 1) or its transcription activator *Nhlh2* (nescient helix-loop-helix 2) genes [91]. However, early reports demonstrated reduced expression levels of *Pcsk1* and Nhlh2 genes and a shift in the ratio of active to inactive precursor forms of circulating hormones, e.g., proinsulin-insulin, preproghrelin-ghrelin, an effect which is presumably due to the impairment of the necessary processing pathway [93]. The same group also reported a significant increase of *Npy* and Agr expression in *Snord116del* mice after refeeding. The *Snord116* deletion also leads to an impairment of pancreatic development, resulting in a reduction of pancreatic islet size and a decrease of *insulin 1(Ins1)* and *insulin 2(Ins2)* gene expression [92]. In addition, expression of Pdx1,**Pax6** and *Nkx6-1* transcription factors, which are important for pancreatic development, was downregulated in adult *Snord116del* mice. In the cerebellum of *Snord116del* at postnatal day 30, the mean cell body diameter of Purkinje neurons was reduced by 21%. In the cortex of *Snord116del* mice, the number of diurnal differentially methylated regions was dramatically reduced, with only 3% of regions showing the same rhythmic methylation pattern that is present in wild-type mice [132]. Among those genes that were epigenetically dysregulated in *Snord116del* mice, functional clusters regulating the circadian entrainment, AMPK (AMP-activated Protein Kinase) signaling, stem cell pluripotency, axon guidance, insulin resistance and dopaminergic synapse function were identified. Interestingly, in *Snord116del* mice, altered diurnal methylation of the imprinted *Dlk1-Dio3* locus and upregulation of its maternally expressed genes were reported, suggesting putative “cross-talk” between two imprinted loci [132].

Results obtained from the *Snord116* KO models are in accordance with the current leading hypothesis that the absence of *SNORD116* gene clusters indeed plays a causative role in the early onset of PWS pathogenesis. *SNORD116* genomic regions became a prime focus following the discovery of PWS patients harboring a rare minimal deletion of the *SNORD116* gene cluster (Figure 1A) [34,35,36,37,38,39].

Despite the fact that the IPW-A exons show little sequence conservation among mammals, we cannot completely rule out functional roles of exon-derived large non-protein coding RNAs in the *PWScr* region. Since *SNORD116* genes from this cluster are the only genes from this region that exhibit a high degree of sequence similarities between different mammals, most research is aimed at elucidating their function. However, the *PWScr* region, as part of a long non-protein coding U-UBE3A-AS transcript, also encodes IPW-A- exons; yet, the functional significance of SNORD116 or non-protein coding IPW-A- exons, or the roles of both in the pathogenesis of PWS are yet to be elucidated.

## 12. Snord115 Gene Cluster

**Figure 3:**
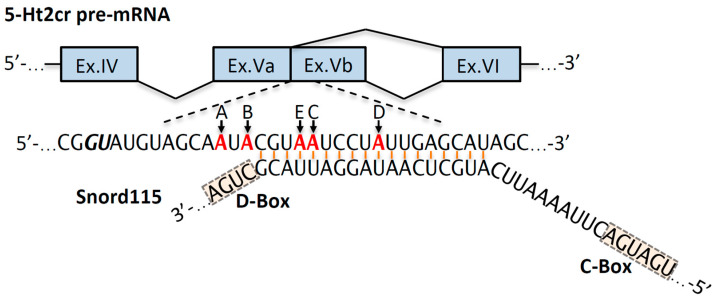
Schematic representation of 5-Ht2cr pre-mRNA exons IV to VI and putative targeting region of Snord115 within exon Vb (drawings are not to scale). Alternative splicing site (*GU*) of 5-Ht2cr pre-mRNA exon Vb is indicated with bold italic letters. A-to-I editing sites A–D are denoted in red and labeled with arrows, accordingly. Snord115 C- and D-boxes are highlighted [128,139].

*SNORD115* is another large, imprinted gene cluster identified within the PWS locus (Figure 1). Historically, it was the first snoRNA with a presumed mRNA target. The antisense element within the snoRNA exhibits an 18 nt long evolutionarily conserved complementarity to the alternatively spliced exon Vb of the 5-HT2C serotonin receptor pre-mRNA that is also subject of posttranscriptional A-to-I editing (Figure 3). The 5-HT2C receptor is part of many complex regulatory networks that, amongst others, have been linked to obesity, feeding behavior, mental state, sleep cycles, autism, neuropsychiatric disorders (e.g., schizophrenia, depression) and neurodegenerative diseases (e.g., Parkinson, Alzheimer) [133,134]. Alternative splicing of exon V of the 5-HT2C receptor pre-mRNA results in an inactive, truncated variant of the receptor. The efficacy of 5-HT2CR G-protein coupling is regulated via posttranscriptional A-to-I editing of the pre-mRNA within the exon Vb region, which generates over 32 different 5-HT2CR mRNA isoforms that collectively modulate the serotonergic signal transduction to varying degrees [135,136]. Ex vivo experiments in HEK293 or Neuro2A cells demonstrated that SNORD115 can interfere with alternative splicing of the 5-HT2C serotonin receptor pre-mRNA. However, this was only observed when the original splice site of pre-mRNA had been mutated for optimal splicing [137]. Studies examining the *PWS-IC^m^*^+/p∆35kb^ mouse model that express barely detectable levels of Snord115, revealed conflicting results. Initially, it was suggested that the absence of Snord115 RNA did not affect alternative splicing of 5-Ht2cr pre-mRNA [83]. However, more recently, the same group reported increased expression of the truncated receptor isoform in the hypothalamus of *PWS-IC^m^*^+/p∆35kb^ mice [138]. In mice, Snord115 is expressed in neurons but is entirely absent from the choroid plexus, an area where 5-Ht2cr mRNA is abundant. RNA deep sequencing analysis of choroid plexus samples from a mouse model with ectopic Snord115 expression revealed that Snord115 is not involved in the regulation of 5-Ht2cr pre-mRNA alternative splicing in vivo [139]. Recently, a long-awaited mouse model harboring the paternal deletion of the *Snord115* gene cluster in the C57BL/6J genetic background was reported (Figure 1C,D; Table 1) [44]. RNA deep sequencing of different brain areas revealed that the lack of Snord115 expression does not alter alternative splicing of 5-Ht2cr pre-mRNA [44]. Likewise, transcriptome analysis of hypothalamus samples from PWS patients did not detect differences in 5-HT2CR pre-mRNA splicing [140].

In addition, potential SNORD115 functions in the regulation of 5-Ht2cr pre-mRNA posttranscriptional A-to-I editing were explored. Ex vivo experiments in cell culture demonstrated that Snord115 can interfere with RNA editing if an atypically nucleolar-localized mRNA substrate is present [141]. The analysis of the “autistic” mouse model, which harbors a paternal duplication of the PWS-locus imprinted genes (patDp/+ mouse) and *PWS-IC^m^*^+/p∆35kb^ mice reported an increase of A-to-I editing of 5-Ht2cr pre-mRNA [83,142]. However, both studies lack sufficient sequencing depth and are controversial due to differences in Snord115 expression levels (~2-fold increase in patDp/+ mice and almost no expression in *PWS-IC^m^*^+/p∆35kb^ mice) [83,142]. The analysis of mouse models with ectopic expression of *Snord115* in the choroid plexus suggested the formation of a double-stranded structure between Snord115 and exon Vb of 5-Ht2cr pre-mRNA, which may also be subject to ADAR (Adenosine deaminases acting on RNA)-mediated A-to-I editing, i.e., similar to the intramolecular duplex formed by exon Vb and the downstream intron of 5-Ht2cr pre-mRNA [139]. Consequently, analysis of 5-Ht2cr pre-mRNA revealed only a modestly reduced A-to-I editing at major sites, questioning the overall biological significance of the Snord115-5Ht2c receptor pre-mRNA interaction and its contribution to PWS pathophysiology [139]. A mouse model with a paternal deletion of the *Snord115* gene cluster also revealed only modest alterations in 5-Htr2cr mRNA editing profiles [44]; moreover, differences were detected in a brain region-specific manner. The functional significance of these minor changes remains to be resolved, as mice showed no obvious phenotypic abnormalities, they bred normally, displayed normal growth patterns and energy balance on either a normal chow or high fat diet. There were no noticeable differences in social or emotional behavioral phenotypes associated with altered 5-Htr2c receptor regulation [44]. Therefore, a functional interaction of *Snord115* with pre-mRNA or mRNA targets, should be considered fortuitous, until there is sound in vivo evidence [143]. This stretch of complementarity is rather testimony to the manner in which sequences of genomes evolve, namely by duplicating existing genetic material and deletions and not by out of the blue de novo generation. As a result, the sequence of genomes is much less complex than theoretically possible, alone due to the presence of at least 50% repetitive elements, e.g., in humans [144].

## 13. PWS Compensation Models

Genetically modified mouse models and the identification of PWS patients with rare small deletions helped to pinpoint the minimal PWS critical region (*PWScr*) to the *SNORD116*/*IPW-A* genomic region. However, an important question remained unanswered. Is the deletion of genomic DNA harboring unknown regulatory elements or is the lack of non-protein coding RNA expression causative of PWS in patients? Therefore, additional mouse models were generated, in which non-protein coding RNA(s) of *PWScr* was re-introduced into expression-lacking animals that exhibited a typical PWS-like phenotype. As the *SNORD116* genes within the *PWScr* region exhibit the highest degree of sequence similarity between different mammalian species, most effort had been devoted to understanding their functions. Two *Snord116* transgenic mouse lines expressing snoRNA embedded in introns of different host genes were generated (Table 1). The first model contained a single copy transgenic *Snord116* within nucleolin intron 11 driven by a neural-specific enolase promoter. This transgene was crossed with a PWS knockout mouse line containing a deletion from *Snrpn* to *Ube3a,* encompassing the complete PWScr [142]. Expression from the transgene failed to rescue either the neonatal lethality or growth retardation of mice with the paternal inheritance of the deletion. These negative results were explained by potentially insufficient expression levels of the single copy gene compared to the highly abundant endogenous Snord116 RNA transcribed from a cluster containing over 66 repeats.

The second model (TgSnord116) was generated by expressing two copies of mouse *Snord116* and one copy of rat *Snord116* from the introns of an *eGFP* host gene [97]. Transgenic mice were crossed with a *PWScr^m+/p−^* mouse model, resulting in *PWScr^m+/p−^TgSnord116* animals. Transgene expression of *Snord116* did not compensate the growth retardation phenotype observed in the *PWScr^m+/p−^* model [97]. A possible explanation could be the tissue-specific expression of the transgenic *eGFP* construct, which was absent in thalamus, hypothalamus, midbrain and pons of *PWScr^m+/p−^TgSnord116* mice [97]. Because dysregulation of the hypothalamic endocrine system is associated with PWS in humans, one would expect that absence of Snord116 expression in this tissue could contribute to growth retardation in mice [145,146]. Consequently, the important question of the functional significance of Snord116 in PWS still needed to be addressed by generating compensatory transgenic animals that express Snord116 in the same brain regions as wild-type mice.

In an attempt to achieve a sufficient expression level of transgenic *Snord116* in the *Snord116del*^m+/p−^ knockout model, a transgene carrying multiple copies of *Snord116* and *Ipw-A* exons was designed to mimic the original gene organization (Table 1) [98]. The transgene construct contained three complete *PWScr* repeat units under the control of a cytomegalovirus (CMV) promoter. Each repeat contained an intron located *Snord116* copy embedded by *Ipw-A* exons. Pronuclear injection of fertilized oocytes with the engineered DNA construct resulted in transgenic mice putatively with head-to-tail donor DNA integration, which often occurs during knock-in or transgene generation [147,148]. The construct was integrated nine times in the genome, resulting in a total of 27 copies of *Snord116* [98]. The insertion was identified at chromosomal region 7qE3 about 47 Mb away from the *PWScr* region. The resulting transgene failed to express *Snord116* and *Ipw-A* exons on a *Snord116del*^m+/p−^ knockout background and hence, to rescue the growth retardation phenotype [98].

In an attempt to re-introduce Snord116 RNAs into the hypothalamus of adult *Snord116del* mouse models (*Snord116del*^m−/p−^) at different ages, an AAV vector expressing a single *Snord116* copy was injected into *Snord116del*^m−/p−^ male mice (Table 1) [100]. Apparently, no significant effects were observed, except for a slight increase in energy expenditure when compared to the vehicle-injected *Snord116del*^m−/p−^ control mice, as well as a reduced rate of weight gain. The effect of the viral *Snord116* expression in knockout animals was rather low and also highly dependent on the age of treated mice and region of injection, generally favoring younger mice and those that were injected in the mid- (rather than anterior) region of the hypothalamus [100]. However, lack of Snord116 expression quantification from the AAV vector together with putative differences observed at various time points of virus microinjections raised questions about the application and potential efficacy of AAV mediated gene therapy for PWS patients.

An alternative strategy was to inactivate the PWS imprinting center on the maternal chromosome (Table 1). To do this, the effects of maternal transmission of the 4.8 kb *IC* and exon 1 *Snrpn* deletion were studied [99]. Maternal inheritance of the deletion resulted in active expression of both protein coding and non-protein coding genes in the PWS locus from the maternal allele in mice whose expression of the paternal allele was impaired due to either a short 4.8 kb deletion on the IC-center at the paternal *PWS-IC^m∆4.8kb/p∆4.8kb^* allele or due to a large chromosomal deletion from *Snrpn* exon 2 to the *Ube3a* gene—*PWS-IC^m∆4.8kb/p∆S-U^*. Remarkably, the maternal expression was able to rescue the prominent postnatal lethality phenotype in both the small (survival rate *PWS-IC^m+/p∆S-U^*: 56%, *PWS-IC^m∆4.8kb/p∆S-U^*: 96%) and large (*PWS-IC^m+/p∆S-U^*: 0%, *PWS-IC^m∆4.8kb/p∆S-U^*: 96%) deletions. Growth retardation was also compensated and the bodyweights of mice inheriting the additional maternal ∆4.8 kb deletion showed no statistically significant difference from their WT littermates by the age of 6 weeks. This was achieved despite the fact that expression levels of the imprinted genes Snrpn (21%–35%), *Snord116* (8%), *Snord115* (10%), and *Ndn* (28%) from the maternal ∆4.8 kb allele were significantly lower compared to wild-type mice [99].

Another strategy applied was the re-activation of imprinted *PWScr* non-protein coding genes from the maternal allele by insertion of the regulatory *LoxP* cassette 5′- to the Snord116 gene cluster in the *PWScr^m+/p−^* mouse model (Table 1) [97]. The PWScr^m5*′*LoxP**/p−^ mice showed a mild growth delay between postnatal day 7 and 19, but over time, gained more weight than their PWScr^m+/p−^ siblings; weight differences to wild-type littermates became insignificant by postnatal day 21. Although the expression levels of Snord116 and Ipw-A non-protein coding RNAs from the maternal allele were reduced by 7.5 and 12-fold, respectively, it apparently was sufficient to rescue the growth retardation phenotype. The inserted 5′*LoxP* cassette in PWScr^m5*′*LoxP**/p−^ mice results in ubiquitous transcriptional activation of *Snord116* and *Snord115* gene clusters from the maternal allele. Expression of Snord64 RNA and protein coding genes at the PWS locus was not perturbed. Although Snord116 was detected in the glial cells of PWScr^m5*′*LoxP**/p−^ mice, the overall brain areas with neuronal snoRNA expression between KI and wild type mice was quite similar [97]. This was the first experimental evidence showing that expression of non-protein coding RNAs is primarily causative of growth retardation in mice and potentially PWS in patients. However, the functional significance of the Snord116 or long RNAs consisting of alternatively spliced Ipw-A exons in the *PWScr* locus or both RNAs is yet to be investigated [97].

## 14. Conclusions

Despite over two decades of investigations, we still lack an in vivo animal model that adequately displays the entire spectrum of PWS symptoms, especially during the second phase that includes obesity. The data supporting a hyperphagic, adult-onset obesity phenotype in mice remains weak at best, with researchers reporting conflicting results despite having used the same knockout strain [43,91]. While some of the conflicting results could be explained by the different methods used to normalize food consumption to respective body weight, this is a far cry from the late-stage morbid obesity observed in patients. This not only shapes the public perception of the disease; it is also the main underlying factor for increased mortality [20].

In general, the differences between various animal models that affect the same gene must also be scrutinized. The use of experimental animals with various genetic backgrounds is known to lead to significant phenotypic differences (see for example [149]). There are experimental, even interpretational variances between laboratories leading to reports of different phenotypes. In different models of gene depleted animals, the target gene is only partially inactivated. If this results, for example, in the expression of a defective (e.g., truncated) gene product, the latter may convey “side” effects. Alternatively, a truncated gene product (or one “rescued” by spicing) still might be functional to various degrees. Finally, off target events, e.g., during propagation in cell culture is a scary but rarely recognized possibility of observing different phenotypes.

Modelling of PWS in mice is complicated by the complexity of the affected region itself, which spans more than 1.5 Mb and contains several genes, quite a few with an associated phenotype of their own. At least for the protein-coding genes, the sequence similarity between mice and humans seems to correlate with functional similarity, as the respective mouse models display a comparable phenotype [33]. The vast majority of human patients are affected by a disruption of these genes in addition to loss of the functionally elusive non-protein coding RNAs and transcripts. The existing mouse models led to a better understanding of the underlying pathophysiological mechanisms and some were tested within a number of ongoing pharmacological trials in search of therapeutic agents [150].

For the non-protein coding transcripts, it is more complicated still. Their mechanistic function is unknown, although all insights gained thus far suggest that they are of importance in both species [151]. Since even rare, small deletions in human patients encompassing nothing more than non-protein coding genes results in the manifestation of core symptoms typical to PWS, further research focusing on the PWScr region with emphasis on the encoded RNAs and transcripts should eventually elucidate a causative mechanism of the disease [34,35,36,37,38,39].

The first generation of compensatory models are in agreement with the identification of the minimal critical region responsible for the PWS phenotype in mice. One of the important insights gained from those models is that transcript localization, proper processing and quantity—but only to a certain degree [97]—are of vital importance. This should be taken into account for prospective models; for example, the natural organization of respective genes should be mimicked as a tandemly repetitive cluster. Moreover, the successful re-activation of the imprinted maternal allele proved to be an option if and when the necessary genome-editing methods are refined enough and deemed save for human application. This could become a feasible therapeutic strategy and an elegant solution for all patients regardless of respective cause, i.e., deletion or maternal uniparental disomy (UPD), since at least one functional allele is always present.

## Figures and Tables

**Table 1:** PWS mouse models and involved genes.

Gene(s) of Interest.	Name, Aliases	Phenotype	References
*all* (UPD Chr 7)	T(7;18)50H/+ (JAX001816, https://www.jax.org/strain/001816, accessed on 20 March 2021)	postnatal lethality (100%) growth retardation	[55] *
*all* (6.8 Mb deletion)	*PWS*^∆LMP2A^ *Tg*^PWS/AS(del)^ Del(7Herc2-Mkrn3)13FRdni/+	fetal growth retardation postnatal growth retardation neonatal lethality (100%) reduced movement irregular respiratory rate hypoglycemia pancreatic apoptosis insulin ↓, glucagon ↓ corticosterone ↑, ghrelin ↑	[40] * [56,57,58]
*Frat3*	*Frat3^lacZ^* *Peg12* ^tm1Brn^	none	[30] *
*Mkrn3*	*Mkrn3* ^m+/p−^	lower weight from P45 earlier onset of puberty GnRH1 ↑	[54] *
*Magel2*	*Magel2*^m+/p−^ *Magel2* KO C57BL/6-*Magel2^tm1Stw^*/J (JAX009062, https://www.jax.org/strain/009062, accessed on 20 March 2021)	*116HG* expression ↓ postnatal lethality (~10%) reduced weight until P28 body fat ↑ lean mass ↓ bone mineral density ↓ impaired glucose homeostasis impaired cholesterol homeostasis insulin resistance leptin resistance dopamine ↓, serotonin ↓ adiponectin ↑ corticosterones ↑ oxytocin ↓ different feeding behavior less active anxiety impaired social behavior delayed onset of puberty progressive infertility	[53] * [59,60,61,62,63,64,65,66,67,68,69,70,71,72]
*Magel2*	*Magel2* ^m+/p−^ *Magel2* ^tm1.1Mus^	postnatal lethality (~50%) weak suckling oxytocin ↓, orexin-A ↓ abnormal social behavior impaired learning ability	[52] * [73]
*Magel2* (overexpression of truncated protein)	*CAG-trMagel2*	neonatal lethality (100%) small body size, poor suckling	[74] *
*Necdin*	*Ndn* ^m+/p−^ *Ndn* ^tm1Alb^	none	[51] *
*Necdin*	*Ndn^tm2Stw^* B6.129S1(Cg)-Ndn^tm2Stw/J^ (JAX009089, https://www.jax.org/strain/009089, accessed on 20 March 2021)	postnatal lethality (80–95% C57BL/6 and 25% FVB) respiratory distress	[48] * [75,76]
*Necdin*	*Ndn*^m+/p−^ B6.129S2-Ndn^tm1.1Mus^	postnatal lethality (21–31%) respiratory distress oxytocin ↓ serotonin ↓	[50] * [77,78]
*Necdin*	*Ndn* ^m+/p−^ *Ndn* ^tm1Ky^	respiratory abnormalities DRG neuron apoptosis ↑ pain sensitivity ↓ noradrenergic excitability ↓	[49] * [79]
Necdin	*Ndn*^m+/p−^ *Necdin* KO *necdin* ^m+/p−^	unstable circadian rhythm	[80] *
*Snurf/Snrpn* *Snord116* *IPW* *Snord115* *Ube3a*	*Snrpn*-*Ube3a* deletion Del(7Ube3a-Snrpn)1Alb	neonatal lethality (80%) growth retardation hypotonia	[41] *
*all* (IC deletion)	*PWS-IC*^∆35kb^ *Snrpn*^tm2Cbr^ *∆PWS-IC* *PWS-IC^del^* *PWS-IC^del35kb^* B6.129-*Snrpn^tm2Cbr^*/J (JAX012443, https://www.jax.org/strain/012443, accessed on 20 March 2021)	neonatal lethality (40–90% depending on background) growth retardation hypotonia decreased locomotive ability abnormal behavior ghrelin ↑ increased food consumption food-seeking behavior ↑	[45] * [81,82,83,84,85,86]
*all* (IC deletion)	*PWS-IC* ^m+/p del4.8kb^ *PWS-IC* ^∆4.8^ *Snrpn* ^tm2Alb^	neonatal lethality (40%) growth retardation	[46] *
*all*, except *Snrpn, Snord64*, *116, 115* (IC deletion)	*PWS-IC* ^Hs^ *Snrpn* ^tm1Kaj^	neonatal lethality (47% C57BL/6J and 16% 129S1/Sv) growth retardation feeding difficulties	[87] *
*all* (IC deletion)	PWS-IC^m+/p∆6kb^ PWS-IC^∆6kb^ B6.129S1-*Snrpn^tm2.1Kaj^*/J (JAX018395, https://www.jax.org/strain/018395, accessed on 20 March 2021)	neonatal lethality (100%) growth retardation feeding difficulties	[47] *
*Snord116*/*IPW*	*PWScr*^m+/p−^ Del(7Ipw-Snord116)1Jbro (distributed by TRAM Münster)	neonatal lethality (15%) growth retardation *pOx* ↑, *Peg3* ↑ decreased gray-matter volume altered sleep profile altered body temperature	[42] * [88,89]
*Snord116*/*IPW*	*Snord116del* *Snord116*^tm1Uta^ *Snord116^+/−P^* B6(Cg)-Snord116^tm1.1Uta^/J (JAX008149, https://www.jax.org/strain/008149, accessed on 20 March 2021)	growth retardation Igf1 ↓ ghrelin ↓ impaired pancreatic development altered diurnal methylation increased anxiety altered respiratory exchange rate resistant to obesity	[43] * [90,91,92,93,94]
*Snord116*/*IPW* (homozygous)	*Snord116* ^m−/p−^ *Snord116* ^−/−^	growth retardation fat mass ↓ increased food consumption altered diurnal activity profile resistant to obesity altered hypothalamic signaling	[95] *
*Snord116*/*IPW* (only *Npy*^+^ Neurons)	*Snord116*^lox/lox^/NPY^cre/+^ (JAX008118, https://www.jax.org/strain/008118, accessed on 20 March 2021)	growth retardation fat mass ↓ increased food consumption altered diurnal activity profile altered hypothalamic signaling	[95] *
*Snord116*/*IPW* (adult-onset)	*Snord116* deletion	reduced food consumption insulin resistance	[96] *
*Snord116*/*IPW* (adult-onset)	*AAV-Snord116del^m^*^+/p−^ *Snord116*^fl^AAV-Cre	increased food consumption bodyweight ↑ bodyfat percentage ↑	[91] *
*Snord115*	*Snord115*-deficient	brown adipose tissue ↑ modest alterations in 5-Htr2cr mRNA A-to-I editing	[44] *
*Snord116* (single copy transgene)		no effect on phenotype	[43] *
*Snord116* (transgene 2 mouse, 1 rat copies)	*PWScr^m+/p−^TgSnord116*	no effect on phenotype	[97] *
*Snord116* (transgene 27 copies)		no effect on phenotype	[98] *
*all* (biallelic IC deletion)	*PWS-IC^m∆4.8kB/p∆4.8kB^* *PWS-IC^m∆4.8kB/p∆S-U^*	rescue of postnatal lethality rescue of growth retardation	[99]
*Snord116/IPW* (maternal IC deletion)	*PWScr*^m*5′LoxP*/p−^ (distributed by TRAM Münster)	Rescue of growth retardation in adult mice alterations in 5-Htr2cr mRNA A-to-I editing in the choroid plexus.	[97] *
Snord116 (AAV-mediated)	*Snord116del*^m−/p−^AAV-*Snord116*	energy expenditure ↑ rate of weight gain ↓	[100] *

Original publications are marked by *, up- and downregulation of physiological parameters is represented by arrows (↑ and ↓).
